# The Associations of *Trans*-3′-Hydroxy Cotinine, Cotinine, and the Nicotine Metabolite Ratio in Pediatric Patients with Tobacco Smoke Exposure

**DOI:** 10.3390/ijerph20095639

**Published:** 2023-04-25

**Authors:** E. Melinda Mahabee-Gittens, Georg E. Matt, Roman A. Jandarov, Ashley L. Merianos

**Affiliations:** 1 Division of Emergency Medicine, Cincinnati Children’s Hospital Medical Center, University of Cincinnati College of Medicine, Cincinnati, OH 45229, USA; 2 Department of Psychology, San Diego State University, San Diego, CA 92123, USA; 3 Division of Biostatistics and Bioinformatics, Department of Environmental and Public Health Sciences, University of Cincinnati College of Medicine, Cincinnati, OH 45267, USA; 4 School of Human Services, University of Cincinnati, Cincinnati, OH 45221, USA

**Keywords:** cotinine, *trans*-3′-hydroxy cotinine, biomarker, children, tobacco smoke exposure

## Abstract

(1) Background: *Trans*-3′-hydroxy cotinine (3HC) and cotinine (COT) are tobacco smoke exposure (TSE) biomarkers and the 3HC/COT ratio is a marker of CYP2A6 activity, an enzyme which metabolizes nicotine. The primary objective was to assess the associations of these TSE biomarkers with sociodemographics and TSE patterns in children who lived with ≥1 smoker. (2) Methods: A convenience sample of 288 children (mean age (SD) = 6.42 (4.8) years) was recruited. Multiple linear regression models were built to assess associations of sociodemographics and TSE patterns with urinary biomarker response variables: (1) 3HC, (2) COT, (3) 3HC+COT sum, and (4) 3HC/COT ratio. (3) Results: All children had detectable 3HC (Geometric Mean [GeoM] = 32.03 ng/mL, 95%CI = 26.97, 38.04) and COT (GeoM = 10.24 ng/mL, 95%CI = 8.82, 11.89). Children with higher cumulative TSE had higher 3HC and COT ($\hat{\beta }$ = 0.03, 95%CI = 0.01, 0.06, *p* = 0.015 and $\hat{\beta }$ = 0.03, 95%CI = 0.01, 0.05, *p* = 0.013, respectively). Highest 3HC+COT sum levels were in children who were Black ($\hat{\beta }$ = 0.60, 95%CI = 0.04, 1.17, *p* = 0.039) and who had higher cumulative TSE ($\hat{\beta }$ = 0.03, 95%CI = 0.01, 0.06, *p* = 0.015). Lowest 3HC/COT ratios were in children who were Black ($\hat{\beta }$ = −0.42, 95%CI = −0.78, −0.07, *p* = 0.021) and female ($\hat{\beta }$ = −0.32, 95%CI = −0.62, −0.01, *p* = 0.044). (4) Conclusion: Results indicate that there are racial and age-related differences in TSE, most likely due to slower nicotine metabolism in non-Hispanic Black children and in younger children.

## 1. Introduction

Despite recent decreases in combustible tobacco use in the United States (U.S.) [1], rates of tobacco smoke exposure (TSE) in children remain high. Overall rates of TSE are approximately 40% in 3–11 year-old children in the U.S, but TSE rates are even higher in children who: are non-Hispanic Black race/ethnicity, live in low-income households, and live in rented homes [2]. When examining factors associated with levels of TSE in nonsmokers, the use of TSE biomarkers can provide important insights into factors associated with TSE levels, such as sociodemographic characteristics and home smoking patterns [3]. Cotinine (COT) is a nicotine metabolite and it is a commonly used TSE biomarker [4]. Prior research indicates that up to 31% of children who live with smokers who seek care in emergency settings have high biochemically confirmed TSE levels that are equivalent to that of active smokers [5,6,7,8]. COT is measurable in the urine, saliva, blood, hair, and nails of exposed children who live with smokers and nonsmokers [3], and it has a half-life of approximately 16 h [5,9]; thus, COT levels reflect recent TSE.

The metabolism of nicotine to COT primarily occurs in the liver by the cytochrome P450 (CYP) enzyme CYP2A6 [10,11]. However, several factors affect the metabolism of nicotine to COT, including race, ethnicity, sex, age, and genetic polymorphisms in the *CYP2A6* gene [4,11,12,13]. Some of the CYP2A6 polymorphisms result in slower metabolism of nicotine in exposed individuals [11], since CYP2A6 activity is correlated with rates of nicotine clearance [14]. These factors may result in varying COT levels in children who are exposed to the same amount of tobacco smoke [5,15].

COT is further metabolized by P450 CYP2A6 to *trans*-3′-hydroxy cotinine (3HC) [16]. 3HC may be a more sensitive biomarker of low-level nicotine exposure compared to COT [17]. When compared to urinary COT, urinary 3HC is 2–4 times more concentrated and has a similar half-life when generated from COT [18,19,20]. The determination of levels of both COT and 3HC metabolites in nonsmoking children may be helpful because the ratio of 3HC to COT, also called the nicotine metabolite ratio (i.e., 3HC/COT), correlates with nicotine metabolism mediated by CYP2A6. Thus, the nicotine metabolite ratio has been used as a measure of the variability in P450 2A6 activity and as a measure of the efficiency of the metabolic processes that result in the conversion of COT to 3HC [14,16,21].

Previously published research on adult smokers [22,23,24] and nonsmoking children with TSE indicate that the nicotine metabolite ratio is higher in non-Hispanic Whites compared to non-Hispanic Blacks [17]. These nicotine metabolite ratio differences could potentially explain why some studies have found that Black children have higher cotinine levels than White children, even after controlling for the amount of TSE [25,26]. However, there is limited research that has examined and compared the nicotine metabolite ratio in tobacco smoke-exposed children of varying sociodemographic backgrounds.

Thus, in this paper, we examined 3HC, COT, 3HC + COT sum, and the nicotine metabolite ratio (3HC/COT) in a sample of 0–17-year-old children who lived with tobacco smokers. Our primary objective was to assess the associations of COT and 3HC in individual, sum, and ratio forms with child sociodemographics and TSE patterns. Based on the extant literature, we hypothesized that we would observe higher levels of 3HC, COT, and 3HC + COT sums but lower 3HC/COT ratio levels in children who were non-Hispanic Black compared to non-Hispanic White children, independent of other child sociodemographics and TSE patterns.

## 2. Materials and Methods

**Figure 1:**
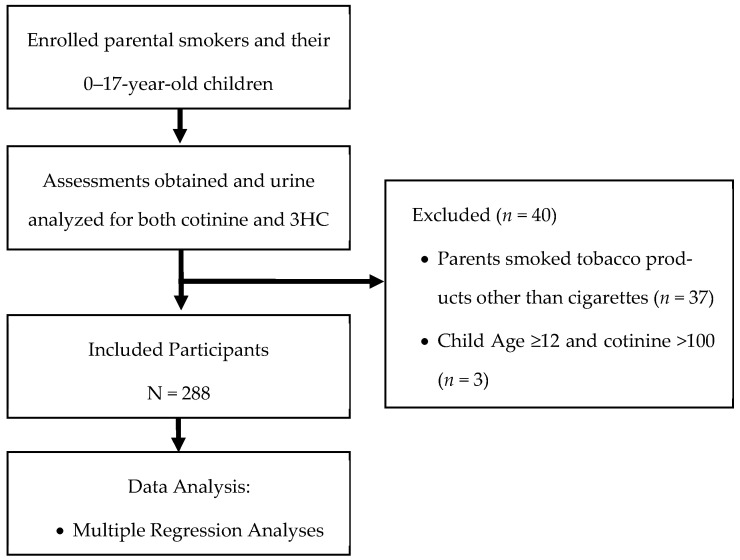
Flow of participants through study.

We analyzed data from a convenience sample of child and parent dyads who were part of a two-group, randomized controlled trial of a parental tobacco cessation trial (“Healthy Families” (clinicaltrials.gov: NCT02531594)) [27]. Study eligibility criteria were as follows: (1) pediatric patients, age 0–17 years old, who were being treated in the pediatric emergency department or urgent care at a midwestern children’s hospital in the U.S., (2) patients presented with a potential TSE-related chief complaint (e.g., rhinorrhea, difficulty breathing), (3) patients lived with a parent who smoked combustible cigarettes only, and (4) patients provided a urine sample during their emergency department or urgent care visit that was analyzed for 3HC or COT. Pediatric patients were excluded if they: (1) lived with a parent who smoked combustible cigars only, as the overall Healthy Families cohort had a low number of child participants whose parents only smoked combustible cigars [28] and because TSE biomarker patterns differ when there is exposure to cigarette smoke compared to cigar smoke [29], or (2) self-reported the use of combustible or noncombustible tobacco or cannabis products. Please see Figure 1. Institutional Review Board approval was obtained (IRB#2015-1914), and parental consent and child assent on children age ≥11 years were obtained prior to conducting any study procedures.

### Self-Reported Child Sociodemographics and TSE Patterns

Parents completed electronic assessments on which they reported child and parent sociodemographics (i.e., child race, ethnicity, age, sex, and insurance type; parent education level; household income level), housing type (i.e., single-family, multi-family, or apartment), and child TSE patterns. Child TSE patterns were assessed with parent reports of: (1) child cumulative TSE, which was the total number of cigarettes smoked around the child by all smokers (e.g., mother, father, siblings, visitors, relatives) in any location (e.g., home, car) in the past week; and (2) home smoking ban (no, yes), which was defined as smoking is never allowed in the home.

## 3. Sample Collection and Analysis

### Urine Collection and Analysis

Urine samples were obtained either via clean catch, in a toilet “hat”, or from diapers in children from which urine was extracted from an inserted pad with a sterile 20 mL syringe and placed into a 14 mL cryovial. Urine was immediately placed on ice and stored at −80 °C until transported on dry ice to the Clinical Pharmacology Laboratory at the University of California at San Francisco. Urine samples were analyzed for free 3HC and free COT by liquid chromatography–tandem mass spectrometry (LC–MS/MS). The lower limit of quantitation (LOQ) for 3HC was 0.10 ng/mL and the LOQ for COT was 0.05 ng/mL [21]. The sum of 3HC + COT and the nicotine metabolite ratio (3HC/COT) were computed based on the molar equivalents of 3HC and COT [17]. The 3HC + COT sum was reported as picomoles of metabolites per milliliter of urine (pmol/mL). Due to the skewed distribution of 3HC, COT, 3HC + COT sum, and 3HC/COT ratio, these four biomarker variables underwent logarithmic transformation prior to analyses.

Since we did not verify children’s self-reported smoking status based on biomarker evidence, all children who were ≥12 years old and had COT levels >100 ng/mL were excluded (*n* = 3; see Figure 1). However, if children <12 years old had COT levels >100 ng/mL (*n* = 4), they were included since prior research shows that children have reached these TSE levels without being primary smokers [30].

## 4. Statistical Analysis

Using R version 4.0.5., we calculated descriptive statistics for all child sociodemographic and TSE pattern variables and report percentages for categorical variables and means (Ms) and standard deviations (SDs) for continuous variables. As mentioned above, all four biomarker variables of interest were log-transformed to address their skewed distributions. We then calculated the geometric means (GeoMs) with 95% confidence intervals (CIs) and medians (Mdns) with interquartile ranges (IQRs) and provide ranges for all four biomarker variables of interest. With a Type 1 error of 0.05, we conducted four separate multiple linear regression analyses to assess the associations of children’s sociodemographics and TSE patterns with the log-transformed urinary biomarker response variables of (1) 3HC, (2) COT, (3) 3HC + COT sum, and (4) 3HC/COT ratio. For each individual model, we present the model fit statistics (R^2^ and *p*-value) and $\hat{\beta }$ and 95%CIs. Based on the results of the four main multiple linear regression models, we conducted sub-analyses to assess the potential interaction effects of the explanatory variables that were significant in these models [31]. Therefore, we also conducted four multiple linear regression models while adding potential interaction terms between child race and child age, sex, and cumulative TSE patterns according to the four log-transformed response variables. For each individual model that includes interaction terms, we also present the model fit statistics (R^2^ and *p*-value) and $\hat{\beta }$ and 95%CIs.

## 5. Results

### 5.1. Child Sociodemographics and TSE Patterns 

The majority of children were Black (58.0%), followed by White (30.9%), other races (9%), and unknown race (2.1%); 97.2% were non-Hispanic ethnicity (Table 1). The mean (SD) child age was 6.42 (4.8) years, and 52.4% were male. Most children had public insurance or were self-pay (92.0%). Over half of the children had parents with an education level ≤ high school graduate/equivalent (51.7%), had a household income level ≤ $15,000 (65.9%), and lived in multi-family homes (22.6%) or apartment buildings (34.4%). Children were around a mean (SD) of 10.3 (21.3) cigarettes smoked around the child by all smokers in any location in the past week (Mdn = 5, IQR = 0, 11). Less than half (39.4%) of children lived in a home that had a smoking ban where smoking was never allowed in the home.

### 5.2. Urinary 3HC, COT, 3HC + COT Sum, and 3HC/Cotinine Ratio Levels

All 288 children had detectable urinary 3HC levels ranging from 0.19 to 780.32 ng/mL (GeoM = 32.03 ng/mL, 95%CI = 26.97, 38.04) and detectable urinary COT levels ranging from 0.04 to 169.01 ng/mL (GeoM = 10.24 ng/mL, 95%CI = 8.82, 11.89). Urinary 3HC + COT sum levels ranged from 0.22 to 842.11 (GeoM = 45.13, 95%CI = 38.42, 53.00) and urinary 3HC/COT ratio levels ranged from 0.12 to 34.69 (GeoM = 3.13, 95%CI = 2.84, 3.45); see Table 2.

### 5.3. Association of Child Sociodemographic Characteristics and TSE Patterns with 3HC and COT Levels

The multiple linear regression model assessing the associations between child sociodemographics and TSE patterns and log-transformed urinary 3HC levels showed an overall significant association (R^2^ = 0.157, *p* = 0.048); see Table 3. Children with higher cumulative TSE had higher 3HC levels (*p* = 0.015). No other significant associations of child sociodemographics and TSE patterns with 3HC biomarker levels were found.

The multiple linear regression model assessing the associations between child sociodemographic characteristics and TSE patterns and log-transformed urinary COT levels showed an overall significant association (R^2^ = 0.300, *p* < 0.001); see Table 3. Black children had higher COT levels (*p* < 0.001) than White children, while adjusting for the other child sociodemographics and TSE patterns. Additionally, children who were younger had higher COT levels (*p* = 0.009). Moreover, higher child cumulative TSE was associated with higher COT levels (*p* = 0.013).

The multiple linear regression model assessing the associations between child sociodemographics and TSE patterns and log-transformed urinary 3HC + COT sum levels showed an overall significant association (R^2^ = 0.187, *p* = 0.012); see Table 3. Black children had higher 3HC + COT sum levels (*p* = 0.039) than White children, while adjusting for the other child sociodemographics and TSE patterns. Children with higher cumulative TSE had higher 3HC + COT sum levels (*p* = 0.015).

The multiple linear regression model assessing the associations between child sociodemographics and TSE patterns and log-transformed urinary 3HC/COT ratio levels did not show an overall significant association (R^2^ = 0.132, *p* = 0.133); see Table 3. Black children had lower 3HC/COT ratio levels (*p* = 0.021) compared to White children, while adjusting for the other child sociodemographics and TSE patterns. Additionally, children who were female had lower 3HC/COT ratio levels (*p* = 0.044).

### 5.4. Associations of Child Age and Child Sex and Urinary 3HC, COT, 3HC + COT Sum, and 3HC/COT Ratio Levels Conditional on Child Race

We assessed all potential two-way interactions between child race and the significant sociodemographic covariates from the results of the main multiple linear regression models, which were child age and child sex. In Table 4, we found a main effect of child age in the separate urinary 3HC, urinary COT, and urinary 3HC + COT sum models. Specifically, each independent model showed that younger children had higher 3HC ($\hat{\beta }$ = −0.08, *p* = 0.013), COT ($\hat{\beta }$ = −0.10, *p* < 0.001), and 3HC + COT sum ($\hat{\beta }$ = −0.09, *p* = 0.002) levels. There were statistically significant interaction effects of child Black race*child age found in these three models. Specifically, each independent model showed that Black children who were older had higher 3HC ($\hat{\beta }$ = 0.08, *p* = 0.044), COT ($\hat{\beta }$ = 0.09, *p* = 0.008), and urinary 3HC + COT sum ($\hat{\beta }$ = 0.09, *p* = 0.019) levels. However, no main or interaction effects of child race and child age were found in the urinary 3HC/COT ratio model.

Concerning child race and child sex, main effects of child race were only found in the separate urinary COT and urinary 3HC + COT sum models. Specifically, both independent models showed that Black children had higher COT ($\hat{\beta }$ = 0.74, *p* = 0.002) and 3HC + COT sum ($\hat{\beta }$ = 0.55, *p* = 0.034) levels, and children of other races ($\hat{\beta }$ = 0.74, *p* = 0.046) had higher urinary COT levels. However, no child race*child sex interaction effects were found in any of the four models; see Table 4.

### 5.5. Associations of Child Cumulative TSE and Urinary 3HC, COT, 3HC + COT Sum, and 3HC/COT Ratio Levels Conditional on Child Race

We assessed potential two-way interactions between child race and the significant child TSE pattern variable of cumulative TSE from the results of the main multiple linear regression models. In Table 5, we found a main effect of child race in the separate urinary 3HC, urinary COT, urinary 3HC + COT sum, and urinary 3HC/COT ratio models. Specifically, Black children had higher urinary 3HC ($\hat{\beta }$ = 0.73, *p* = 0.001), urinary COT ($\hat{\beta }$ = 1.03, *p* < 0.001), and urinary 3HC + COT sum ($\hat{\beta }$ = 0.81, *p* < 0.001) levels, but lower urinary 3HC/COT ratio levels ($\hat{\beta }$ = −0.30, *p* = 0.020). Children of other races ($\hat{\beta }$ = 0.83, *p* = 0.020) also had higher urinary COT levels. We also found main effects of child cumulative TSE in the separate urinary 3HC, urinary COT, and urinary 3HC + COT sum models. Specifically, each independent model showed that children with higher cumulative TSE had higher 3HC ($\hat{\beta }$ = 0.03, *p* < 0.001), COT ($\hat{\beta }$ = 0.03, *p* < 0.001), and 3HC + COT sum ($\hat{\beta }$ = 0.30, *p* < 0.001) levels.

We found statistically significant interaction effects of child Black race*child cumulative TSE in the separate urinary 3HC, urinary COT, and urinary 3HC + COT sum models. Specifically, each independent model showed that Black children who had higher cumulative TSE had lower urinary 3HC ($\hat{\beta }$ = −0.03, *p* = 0.005), urinary COT ($\hat{\beta }$ = −0.02, *p* = 0.002), and urinary 3HC + COT sum ($\hat{\beta }$ = −0.03, *p* = 0.003) levels. However, no main or interaction model effects for child race and child cumulative TSE were found in the urinary 3HC/COT ratio model; see Table 5.

## 6. Discussion

This study investigated the associations of urinary 3HC, COT, 3HC + COT sum, and the nicotine metabolite ratio (3HC/COT) with children’s sociodemographic characteristics and child TSE patterns in a cohort of children who lived with smokers. Consistent with our hypothesis, we observed higher levels of COT and 3HC + COT sums, but lower 3HC/COT ratio levels in children who were non-Hispanic Black compared to children who were non-Hispanic White race/ethnicity, independent of other child sociodemographic and TSE patterns. Regarding COT, other prior research has also found that COT levels are higher in children who are of Black race and who are younger [2,7,32]. These higher COT levels may be due to slower metabolism secondary to genetic polymorphisms in the CYP2A6 gene in these child groups [4,11,12,13].

As expected, children with higher cumulative TSE had higher 3HC and COT levels. However, the results of the multiple linear regression models, which indicate that there are differences in 3HC + COT sums and 3HC/COT ratio levels observed in Black children compared to White children, are important findings that add to the literature. Previously, Matt et al. [17] reported that COT, 3HC, and 3HC + COT sum levels are strong and stable biomarkers of TSE, and that the 3HC/COT ratio is higher in younger children and lower in non-Hispanic Black children. Our study expands on this and other related work [12,17,22] by including a larger sample of children who were highly exposed to tobacco smoke, over half of which were of non-Hispanic Black race/ethnicity.

The finding that Black children had lower 3HC/COT ratios even when the models controlled for TSE patterns adds credence to past work which suggests that the same levels of COT and 3HC are not equivalent to the same exposure levels in Black children compared to White children. In other words, when Black children and White children have the same COT levels, they may not be exposed to the same number of cigarettes and related tobacco smoke pollutants. This is because Black children with TSE have slower metabolism of nicotine, as suggested in this study and in prior work [12,17,22].

This is also consistent with previously published research on adult smokers, which indicates that Black smokers have a lower nicotine metabolite ratio [33,34,35] and higher levels of TSE biomarkers compared to White adult smokers [36,37]. Consequently, when Black children inhale the same amount of tobacco smoke pollutants as White children, given the slower metabolism of some of these toxicants (e.g., nicotine, TSNAs) [25,38,39], it is possible that Black children may be more vulnerable to exposure-related harms since these pollutants stay in their bodies longer. Thus, these results suggest that although Black children may have similar TSE biomarker levels as White children, they may have higher tobacco-related morbidity in the future. These findings may partially explain why tobacco-related health disparities are higher in Black adult smokers who smoke fewer cigarettes and who may have started smoking at a later age than their White adult counterparts [40,41,42]. Future research should consider assessing race-related interaction terms to account for the differential validity of cotinine levels among racially diverse children. More research is encouraged to determine whether the clinically relevant cotinine biomarker threshold of TSE for Black children should be lowered compared to White children. Optimal cotinine cutpoints have been suggested for U.S. adolescents and adults based on racial/ethnic groups, but these cutpoints have not been suggested for children exposed to SHS and THS [15].

Strengths of this study include the examination of a cohort of children who lived with smokers and who had high levels of TSE. Another strength is the examination of objective TSE biomarkers. Limitations of this study include the use of parent-reported child TSE patterns which may be subject to recall biases, and parents may have been unaware of the patterns of tobacco use in household members. However, reported TSE was biochemically confirmed with cotinine and 3HC, which strengthens the results. If parent reports were underestimated, then our findings would be even stronger if this limitation were removed. Second, our results are not generalizable since they are from a convenience sample of a single cohort of children who visited emergency settings at one children’s hospital. Third, since this study was cross-sectional, causal inferences should be made with caution. Future research should include large cohorts of racially/ethnically diverse children from multiple study sites who are followed longitudinally to determine the reproducibility and stability of these findings over time. Fourth, we did not correct 3HC or cotinine by specific gravity or urine creatinine; variations in these values may have affected the results [43]. Finally, future research should assess the amount of time that elapsed between when the child was last exposed to tobacco smoke and when urine was collected as the timing is likely associated with the urinary 3HC/COT ratio levels.

In summary, this study corroborates prior work and indicates that there are racial-related and age-related differences in TSE biomarkers, most likely due to slower nicotine metabolism in non-Hispanic Black children and in younger children. These results suggest that lower CYP2A6 activity in non-Hispanic Black children may impede the detoxication of TSNAs compared to children with higher enzyme activity. These differences in nicotine metabolism may lead to misinterpretations of exposure status and exposure patterns of children in these sociodemographic groups.

## Figures and Tables

**Table 1:** Child Sociodemographics and TSE Patterns.

Characteristic (N = 288)	*n* (%) ^a^
**Child Race**	
Black White	167 (58.0) 89 (30.9)
Other	26 (9.0)
Unknown	6 (2.1)
**Child Ethnicity**	
Non-Hispanic	280 (97.2)
Hispanic	4 (1.4)
Unknown	4 (1.4)
**Child Age, *M* (SD)**	6.42 (4.8)
**Child Sex**	
Male	151 (52.4)
Female	137 (47.6)
**Child Insurance Type**	
Public/self-pay Commercial	265 (92.0) 23 (8.0)
**Parent Education Level**	
≤High school graduate/equivalent	149 (51.7)
≥Some college	139 (48.3)
**Income Level (U.S. Dollars, $)**	
≤$15,000	189 (65.9)
>$15,000	98 (34.1)
**Housing Type**	
Single-Family	124 (43.0)
Multi-Family	65 (22.6)
Apartment	99 (34.4)
**Child Cumulative TSE, ^b^ *M* (SD)**	10.32 (21.3)
**Home Smoking Ban ^c^**	
No	80 (60.6)
Yes	52 (39.4)

Abbreviations: TSE, tobacco smoke exposure; M, mean; SD, standard deviation; Mdn, median; IQR, interquartile range. ^a^ *n* (column %) unless noted otherwise. ^b^ Total number of cigarettes smoked around the child by all smokers in any location in the past week. ^c^ Smoking is never allowed in the home.

**Table 2:** Child Urinary Biomarker Levels.

Urinary Biomarker (N = 288)	GeoM (95%CI)	Mdn (IQR)	Range (Min–Max)
**3HC (ng/mL)**	32.03 (26.97, 38.04)	36.89 (12.81, 97.36)	0.19–780.32
**COT (ng/mL)**	10.24 (8.82, 11.89)	11.82 (4.65, 25.73)	0.04–169.01
**3HC + COT Sum (pmol/mL)**	45.13 (38.42, 53.00)	53.63 (19.00, 121.93)	0.22–842.11
**3HC/COT Ratio**	3.13 (2.84, 3.45)	3.17 (1.85, 5.73)	0.12–34.69

Abbreviations: GeoM, geometric mean; CI, confidence interval; Mdn, median; IQR, interquartile range; Min, minimum; Max, maximum; 3HC, *trans*-3′-hydroyxcotinine; COT, cotinine.

**Table 3:** Multiple Linear Regression Model Results of Child Sociodemographics and TSE Patterns with Urinary 3HC, Urinary COT, Urinary 3HC + COT Sum, and Urinary 3HC/COT Ratio Biomarker Levels.

	Urinary 3HC Model (N = 288)		Urinary COT Model (N = 288)	
**Model Fit Statistics**	R^2^ = 0.157 *p* = 0.048		R^2^ = 0.300 *p* < 0.001	
	$\hat{\beta }$ **(95%CI)**	***p*** **-value ^a^**	$\hat{\beta }$ **(95%CI)**	***p*** **-value ^a^**
**Child Race**				
White	Ref		Ref	
Black	0.49 (−0.12, 1.10)	0.119	**0.92 (0.42, 1.41)**	**<0.001**
Other	0.37 (−0.65, 1.39)	0.481	0.42 (−0.40, 1.24)	0.319
Unknown	0.46 (−1.24, 2.15)	0.600	0.89 (−0.48, 2.26)	0.205
**Child Age**	−0.04 (−0.09, 0.02)	0.229	**−0.06 (−0.11, −0.02)**	**0.009**
**Child Sex**				
Male	Ref		Ref	
Female	−0.18 (−0.71, 0.34)	0.498	0.14 (−0.29, 0.56)	0.533
**Child Insurance Type**				
Commercial	Ref		Ref	
Public/self-pay	0.10 (−0.82, 1.01)	0.836	0.50 (−0.24, 1.24)	0.189
**Parent Education Level**				
≤High school graduate/equivalent	Ref		Ref	
≥Some college	0.01 (−0.54, 0.53)	0.996	−0.13 (−0.56, 0.30)	0.554
**Income Level**				
≤$15,000	Ref		Ref	
>$15,000	−0.29 (−0.90, 0.32)	0.3519	−0.23 (−0.72, 0.26)	0.354
**Housing Type**				
Single-Family	Ref		Ref	
Multi-Family	0.11 (−0.55, 0.78)	0.739	−0.16 (−0.69, 0.38)	0.566
Apartment	0.11 (−0.50, 0.72)	0.722	0.07 (−0.43, 0.56)	0.788
**Child Cumulative TSE ^b^**	**0.03 (0.01, 0.06)**	**0.015**	**0.03 (0.01, 0.05)**	**0.013**
**Home Smoking Ban ^c^**				
No	Ref		Ref	
Yes	−0.51 (−1.12, 0.10)	0.107	−0.46 (−0.95, 0.04)	0.073
	**Urinary 3HC + COT Sum Model ** **(N = 288)**		**Urinary 3HC/COT Ratio Model** **(N = 288)**	
**Model Fit Statistics**	R^2^ = 0.187 *p* = 0.012		R^2^ = 0.132 *p* = 0.133	
	$\hat{\beta }$ **(95%CI)**	***p*** **-value ^a^**	$\hat{\beta }$ **(95%CI)**	***p*** **-value ^a^**
**Child Race**				
White	Ref		Ref	
Black	**0.60 (0.04, 1.17)**	**0.039**	**−0.42 (−0.78, −0.07)**	**0.021**
Other	0.36 (−0.58, 1.29)	0.460	−0.05 (−0.65, 0.54)	0.862
Unknown	0.53 (−1.04, 2.09)	0.510	−0.44 (−1.42, 0.55)	0.387
**Child Age**	−0.05 (−0.10, 0.01)	0.081	0.03 (−0.01, 0.06)	0.108
**Child Sex**				
Male	Ref		Ref	
Female	−0.05 (−0.54, 0.44)	0.841	**−0.32 (−0.62, −0.01)**	**0.044**
**Child Insurance Type**				
Commercial	Ref		Ref	
Public/self-pay	0.20 (−0.64, 1.04)	0.642	−0.40 (−0.93, 0.13)	0.141
**Parent Education Level**				
≤High school graduate/equivalent	Ref		Ref	
≥Some college	−0.06 (−0.55, 0.44)	0.823	0.13 (−0.18, 0.44)	0.416
**Income Level**				
≤$15,000	Ref		Ref	
>$15,000	−0.23 (−0.79, 0.33)	0.427	−0.06 (−0.41, 0.30)	0.754
**Housing Type**				
Single-Family	Ref		Ref	
Multi-Family	0.05 (−0.56, 0.66)	0.866	0.27 (−0.12, 0.66)	0.173
Apartment	0.16 (−0.4, 0.72)	0.579	0.04 (−0.31, 0.40)	0.812
**Child Cumulative TSE ^b^**	**0.03 (0.01, 0.06)**	**0.015**	0.01 (−0.01, 0.02)	0.468
**Home Smoking Ban ^c^**				
No	Ref		Ref	
Yes	−0.49 (−1.05, 0.07)	0.088	−0.05 (−0.41, 0.30)	0.778

Abbreviations: 3HC, *trans*-3′-hydroyxcotinine; COT, cotinine; CI, confidence interval; Ref, reference; TSE, tobacco smoke exposure. ^a^ Bold font indicates statistical significance *p* < 0.05. ^b^ Total number of cigarettes smoked around the child by all smokers in any location in the past week. ^c^ Smoking is never allowed in the home.

**Table 4:** Multiple Linear Regression Model Results of Child Age and Child Sex and Urinary 3HC, COT, 3HC + COT Sum, and 3HC/COT Ratio Levels Conditional on Child Race.

	Urinary 3HC Model (N = 288)		Urinary COT Model (N = 288)		Urinary 3HC + COT Sum Model (N = 288)		Urinary 3HC/COT Ratio Model (N = 288)	
**Child Race and Child Age Models**								
**Model Fit Statistics**	R^2^ = 0.039 *p* = 0.131		R^2^ = 0.112 *p* < 0.001		R^2^ = 0.058 *p* = 0.018		R^2^ = 0.052 *p* = 0.034	
	$\hat{\beta }$	***p*** **-Value ^a^**	$\hat{\beta }$	***p*** **-Value ^a^**	$\hat{\beta }$	***p*** **-Value**	$\hat{\beta }$	***p*** **-Value ^a^**
**Child Race**								
White	Ref		Ref		Ref		Ref	
Black	−0.10	0.755	0.20	0.462	−0.04	0.893	−0.29	0.100
Other	−0.48	0.377	0.04	0.935	−0.42	0.413	−0.52	0.092
Unknown	−0.13	0.895	0.12	0.884	−0.10	0.912	−0.24	0.652
**Child Age**	−0.08	**0.013**	−0.10	**<0.001**	−0.09	**0.002**	0.02	0.211
Child Race*Age Interactions								
Child Black Race*Age	0.08	**0.044**	0.09	**0.008**	0.09	**0.019**	−0.01	0.687
Child Other Race*Age	0.12	0.090	0.08	0.208	0.12	0.081	0.05	0.252
Child Unknown Race*Age	0.18	0.510	0.13	0.536	0.16	0.509	0.04	0.803
**Child Race and Child Sex Models**								
**Model Fit Statistics**	R^2^ = 0.0213 *p* = 0.532		R^2^ = 0.0705 *p* = 0.004		R^2^ = 0.0302 *p* = 0.278		R^2^ = 0.045 *p* = 0.069	
	$\hat{\beta }$	***p*** **-Value ^a^**	$\hat{\beta }$	***p*** **-Value ^a^**	$\hat{\beta }$	***p*** **-Value ^a^**	$\hat{\beta }$	***p*** **-Value ^a^**
**Child Race**								
White	Ref		Ref		Ref		Ref	
Black	0.48	0.081	0.74	**0.002**	0.55	**0.034**	−0.26	0.092
Other	0.35	0.420	0.74	**0.046**	0.42	0.298	−0.39	0.113
Unknown	0.49	0.586	0.92	0.222	0.57	0.492	−0.44	0.380
**Child Sex**								
Male	Ref		Ref		Ref		Ref	
Female	−0.07	0.821	−0.03	0.909	−0.06	0.843	−0.04	0.817
Child Race*Sex Interactions								
Child Black Race*Sex	−0.22	0.581	−0.05	0.872	−0.15	0.675	−0.16	0.459
Child Other Race*Sex	−0.28	0.679	−0.66	0.250	−0.38	0.553	0.38	0.315
Child Unknown Race*Sex	0.22	0.864	−0.23	0.830	0.11	0.926	0.44	0.529

Abbreviations: 3HC, *trans*-3′-hydroyxcotinine; COT, cotinine; Ref, reference. ^a^ Bold font indicates statistical significance *p* < 0.05.

**Table 5:** Multiple Linear Regression Model Results of Child Cumulative TSE and Urinary 3HC, COT, 3HC + COT Sum, and 3HC/COT Ratio Levels Conditional on Child Race.

	Urinary 3HC Model (N = 288)		Urinary COT Model (N = 288)		Urinary 3HC + COT Sum Model (N = 288)		Urinary 3HC/COT Ratio Model (N = 288)	
**Child Race and Child Cumulative TSE Models**								
**Model Fit Statistics**	R^2^ = 0.083 *p* = 0.002		R^2^ = 0.142 *p* < 0.001		R^2^ = 0.098 *p* < 0.001		R^2^ = 0.035 *p* = 0.225	
	$\hat{\beta }$	***p*** **-Value ^a^**	$\hat{\beta }$	***p*** **-Value ^a^**	$\hat{\beta }$	***p*** **-Value ^a^**	$\hat{\beta }$	***p*** **-Value ^a^**
**Child Race**								
White	Ref		Ref		Ref		Ref	
Black	0.73	**0.001**	1.03	**<0.001**	0.81	**<0.001**	−0.30	**0.020**
Other	0.51	0.230	0.83	**0.020**	0.56	0.154	−0.32	0.211
Unknown	1.32	0.185	1.49	0.074	1.36	0.140	−0.17	0.775
**Child Cumulative TSE ^b^**	0.03	**<0.001**	0.03	**<0.001**	0.03	**<0.001**	0.00	0.557
Child Race*Cumulative TSE **Interactions**								
Child Black Race*Cumulative TSE	−0.03	**0.005**	−0.02	**0.002**	−0.03	**0.003**	−0.00	0.724
Child Other Race*Cumulative TSE	−0.00	0.969	−0.02	0.586	−0.01	0.885	0.02	0.480
Child Unknown Race* Cumulative TSE	0.00	0.985	−0.03	0.871	−0.02	0.955	0.03	0.794

Abbreviations: 3HC, *trans*-3′-hydroyxcotinine; COT, cotinine; Ref, reference; TSE, tobacco smoke exposure. ^a^ Bold font indicates statistical significance *p* < 0.05. ^b^ Total number of cigarettes smoked around the child by all smokers in any location in the past.

## Data Availability

The dataset will be made available to other qualified researchers upon reasonable request.
